# Development of a prognostic index based on immunogenomic landscape analysis in glioma

**DOI:** 10.1002/iid3.407

**Published:** 2021-01-27

**Authors:** Haitao Luo, Chuming Tao, Peng Wang, Jingying Li, Kai Huang, Xingen Zhu

**Affiliations:** ^1^ Department of Neurosurgery The Second Affiliated Hospital of Nanchang University Nanchang Jiangxi China; ^2^ East China Institute of Digital Medical Engineering Shangrao Jiangxi China; ^3^ Department of Comprehensive Intensive Care Unit The Second Affiliated Hospital of Nanchang University Nanchang Jiangxi China; ^4^ Institute of Neuroscience Nanchang University Nanchang Jiangxi China

**Keywords:** glioma, immune‐related genes, immunotherapy, prognosis index

## Abstract

**Background:**

Glioma is the most common intracranial tumor. The inflammatory response actively participates in the malignancy of gliomas. There is still limited knowledge about the biological function of immune‐related genes (IRGs) and their potential involvement in the malignancy of gliomas.

**Methods:**

We screened differentially expressed and survival‐associated IRGs, and explored their potential molecular characteristics. Then we developed a prognostic index derived from seven hub IRGs. A prognostic nomogram was built to indicate the prognostic value of the prognostic index and seven IRGs. We characterized the immune infiltration landscape to analyze tumor‐immune interactions. The real‐time quantitative polymerase chain reaction assay was performed to validate bioinformatics results.

**Results:**

The differentially expressed IRGs are involved in cell chemotaxis, cytokine activity, and the chemokine‐mediated signaling pathway. The prognostic index derived from seven IRGs had clinical prognostic value in glioma, and positively correlated with the malignant clinicopathological characteristics. A nomogram further indicated that the prognostic index and seven hub IRGs had clinical prognostic value for gliomas. We revealed that the prognostic index could reflect the state of the glioma immune microenvironment.

**Conclusion:**

This study demonstrates the importance of an IRG‐based prognostic index as a potential biomarker for predicting malignancy in gliomas.

## Graphical abstract

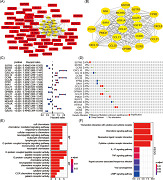

## 1 INTRODUCTION

Glioblastoma (GBM) is the most aggressive intracranial tumor.^1^, ^2^ The standard treatment strategies for GBM composed of precise surgical resection with preoperative imaging, adjuvant chemotherapy, and postoperative radiotherapy.^3^, ^4^, ^5^ However, the overall survival (OS) rates of patients with glioma remain poor following these standard therapies.^6^, ^7^ Hence, a need exists to further explore more advanced treatments for glioma.

Immunotherapy has been extensively studied as a novel perspective treatment for human malignant tumor.^8^, ^9^, ^10^ Compared with chemotherapy and radiotherapy, immunotherapy is more specific for tumor cells without normal cell targeting. For example, an immune checkpoint inhibitor, programmed death 1 (PD 1) and its ligand (PD‐L1), specifically targets activated T‐cells, and shows noteworthy clinical benefits in the treatment of malignant tumors.^11^ However, these immune checkpoint inhibitors can induce rapid responses and extend OS in patients, only a few patients with glioma benefit from immunotherapy. Immunotherapy has yet to demonstrate an objective benefit among all Phase III clinical trials to date.^12^, ^13^

More recently, there is still limited knowledge about the biological function of immune‐related genes (IRGs) and their potential involvement in the malignancy of gliomas. In this study, we sought to explore the clinical implications of IRGs in predicting prognosis and their underling functions as biomarkers for the diagnosis and treatment of gliomas. Our results provide novel insights for the advanced clinical application of immunotherapy and personalized therapy of gliomas.

## 2 MATHODS AND METHODS

### 2.1 Data collection and acquisition

Clinicopathological data of glioma samples from the The Cancer Genome Atlas (TCGA) (*n* = 628) (https://portal.gdc.cancer.gov/), the Chinese Glioma Genome Atlas (CGGA) (*n* = 298) (http://www.cgga.org.cn/) and GSE16011 (*n* = 263) datasets are presented in Table S1. We obtained a list of IRGs from the Immunology Database and Analysis Portal (ImmPort) dataset (https://immport.niaid.nih.gov),^14^ and copy number alterations data from cBioPortal (http://www.cbioportal.org/).^15^ A list of 318 transcription factors (TFs) were obtained from the Cistrome Cancer dataset (http://www.cistrome.org/).^16^ Immune cell infiltration level (B cells, CD8+ T cells, CD4+ T cells, macrophages, neutrophils, and dendritic cells) data in tumors were downloaded from The Tumor Immune Estimation Resource (TIMER) online dataset data set (https://cistrome.shinyapps.io/timer/).^17^

### 2.2 Selected differential expressed and survival‐associated IRGs

The R package “limma” was applied to identify differentially expressed IRGs between low grade glioma (LGG) and GBM. The Gene Ontology (GO) and Kyoto Encyclopedia of Genes and Genomes (KEGG) pathway analyses were performed to explore the underling biological functions of these IRGs using the R package “clusterProfiler,” “enrichplot,” and “ggplot2.” Univariate Cox regression analysis was used to select survival‐associated IRGs using the R package “survival.”

### 2.3 Molecular characteristics of hub IRGs

A hub IRG is a gene that plays a significant role in biological process, which often affects the regulation of other IRGs in related pathways.^18^ In this study, we also systematically analyzed the clinical values of our hub IRGs. To explore the correlation between survival‐related IRGs, we constructed a protein–protein interaction (PPI) network via the string online tool (https://stringdb.org/).^19^ The PPI network was displayed using Cytoscape software v3.6.1.^20^ We performed functional enrichment analysis to explore the potential biological functions of these hub IRGs. We also searched whether some TFs had potential biological functions in regulating these hub IRGs. We selected survival‐related TFs using the R package “limma” and constructed the TFs‐IRGs network between selected TFs and hub IRGs using Cytoscape software.

### 2.4 Construction of a clinical prognostic model

To explore the potential molecular mechanisms of our hub IRGs, we employed the least absolute shrinkage and selection operator (LASSO) Cox regression algorithm to develop a potential prognostic index.^21^, ^22^ Minimum criteria were utilized to define the seven IRGs (SSTR5, CXCL10, CCL13, SAA1, CCL21, CCL27, and HTR1A) and to select the optimal penalization coefficient lambda. The prognostic index was defined using the formula: prognostic index = [SSTR5 expression × (−0.2742)] + [CXCL10 expression × (0.1171)] + [CCL13 expression × (0.087)] + [SAA1 expression ×  (0.084)] + [CCL21 expression × (−0.1978)] + [CCL27 expression × (0.2163)] + [HTR1A expression × (−0.1284)]. This formula was used to calculate a prognostic index for the glioma samples in the TCGA, CGGA, and GSE16011 datasets. We sorted the glioma samples into two subgroups based on the median prognostic index. The receiver operating characteristic (ROC) curve was implemented to assess the prediction efficiency of our prognostic index through the R package “survivalROC.” The nomograms were formulated with R package “rms” and “foreign.” We evaluated the nomograms performance via the concordance index (C‐index) and by comparing nomogram‐predicted with Kaplan–Meier estimates of survival probability. Then, we explored the correlations between clinicopathological features, infiltration of six immune cell types, and prognostic index. Finally, to explore the role our seven selected IRGs played in the malignancy of gliomas, we systematically analyzed their expression in relation to different clinicopathological features (age, gender, World Health Organization [WHO] grade, isocitrate dehydrogenase [IDH] status, and 1p/19q status), and identified related signaling pathways using gene set enrichment analysis.^23^

### 2.5 Real‐time quantitative polymerase chain reaction

Normal brain tissues (NBT) and glioma tissues were obtained from department of Neurosurgery, the second affiliated hospital of Nanchang University during January 2017 to January 2020. The information of the patients was list in Table S2. Total RNA was isolated from the frozen tissue specimens using trizol reagent (Invitrogen) following the manufacturer's instructions. The real‐time quantitative polymerase chain reaction (RT‐qPCR) was performed on The LightCycler 480 Real‐Time PCR System. The seven selected IRGs were, respectively, assayed by qPCR on an Applied Biosystems Real Time Instrument with three steps. For each PCR detection: predenaturation at 95°C for 10 min, followed by 40 cycles at 95°C for 15 s and 60°C for 60 s. The expression levels of genes were measured by the comparative cycle threshold (ΔΔCt) method. The sequences of forward and reverse primers for the seven IRGs were listed in Table S3. All samples were repeated in triplicate. Our experiments were conducted the approval of the Ethics Committee of the second affiliated hospital of Nanchang University.

### 2.6 Statistical analyses

Kaplan–Meier survival curves were generated to compare patient OS in different categories. The LASSO Cox regression algorithm was used for developing a potential prognostic index. The *t* test was applied to evaluate the expression of the seven selected IRGs in relation to different clinical characteristics. The relationship between the prognostic index and different clinical characteristics was determined via univariate and multivariate Cox regression analyses. Statistical analyses were performed using SPSS software 26.0 (SPSS Inc.), R software v3.6.3 (http://www.r-projiect.org/) and Prism 8 (GraphPad Software, Inc.). Data were considered significant at *p* < .05.

## 3 RESULTS

### 3.1 Identification of differentially expressed and survival‐associated IRGs

**Figure 1:**
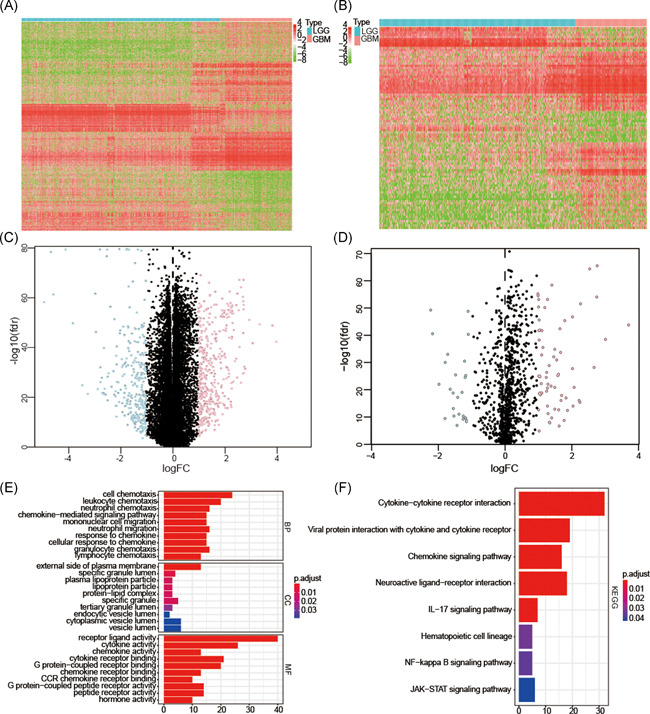
Differentially expressed IRGs and their function. The differentially expressed genes were selected by Heatmap construction (A) and volcano plot (C) between low grade glioma (LGG) and glioblastoma (GBM). Differentially expressed IRGs were also selected by the heatmap (B) and volcano plot (D). Functional annotations of differentially expressed IRGs were obtained by Gene ontology (GO) biological process terms (E) and Kyoto Encyclopedia of Genes and Genomes (KEGG) pathway analyses (F).

To identify survival‐associated IRGs, we first screened 676 differentially expressed genes in the TCGA dataset, including 392 upregulated and 284 downregulated genes (Figures 1A and 1C). Among these genes, 91 differentially expressed IRGs were found, including 66 upregulated and 25 downregulated (Figures 1B and 1D). Using GO analysis, we found these IRGs involved in the external side of the plasma membrane, cell chemotaxis, cytokine activity, chemokine‐mediated signaling pathways, cellular responses to chemokines, and chemokine activity were enriched (Figure 1E). And KEGG pathway analysis further disclosed significant enrichment of IRGs in cytokine‐cytokine receptor interaction, the chemokine signaling pathway, and neuroactive ligand‐receptor interaction (Figure 1F). The inflammatory responses were most frequently implicated. We further confirmed that all of these IRGs were associated with prognosis (*p* < .05) (Table S4).

### 3.2 Selection and exploration of the characteristics of hub IRGs

**Figure 2:**
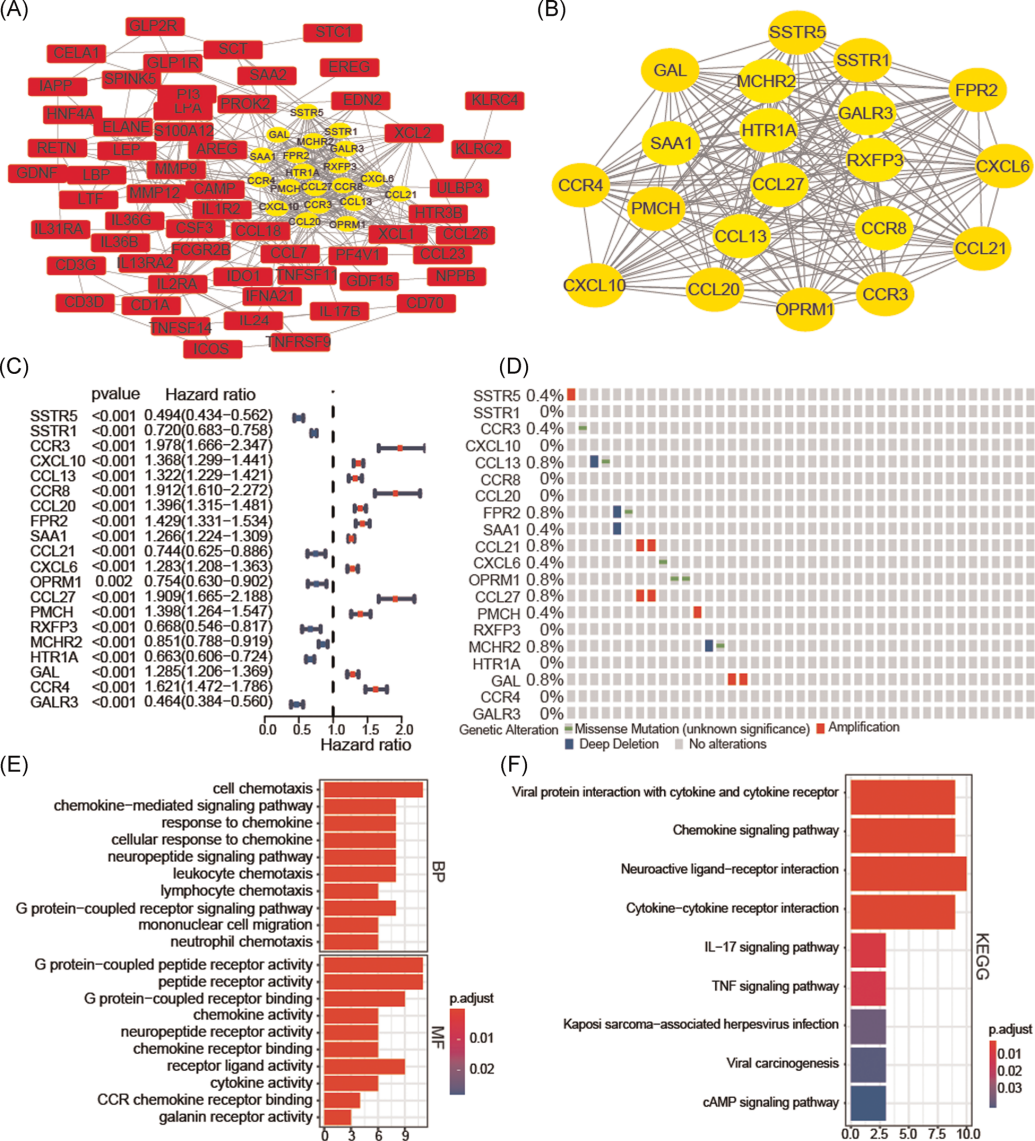
Identification of hub IRGs and exploring their biological function. The protein–protein interaction (PPI) network of 91 survival‐associated IRGs (A) and 20 hub IRGs (B). The forest plot showing the prognostic values of hub IRGs (C). Genetic changes of 20 hub IRGs (D). Functional annotations of hub IRGs were obtained by GO (E) and KEGG (F) pathway analyses. GBM, glioblastoma; GO, Gene Ontology; IRG, immune‐related genes; KEGG, Kyoto Encyclopedia of Genes and Genomes

We defined 20 hub IRGs through the PPI network (Figure 2A,B), including 8 downregulated and 12 upregulated IRGs (Figure 2C). These hub IRGs involved in response to chemokine, cell chemotaxis, chemokine‐mediated signaling pathway, the chemokine signaling pathway, interleukin‐17 signaling pathway, TNF signaling pathway, and cytokine‐cytokine receptor interaction were the most frequently implicated (Figure 2E,F). The frequency of genetic alterations in our hub IRGs was very low (<0.8%; Figure 2D), revealing that the different expression levels of hub IRGs were not caused by genetic alterations.

### 3.3 Establishment of the TFs‐IRGs network

**Figure 3:**
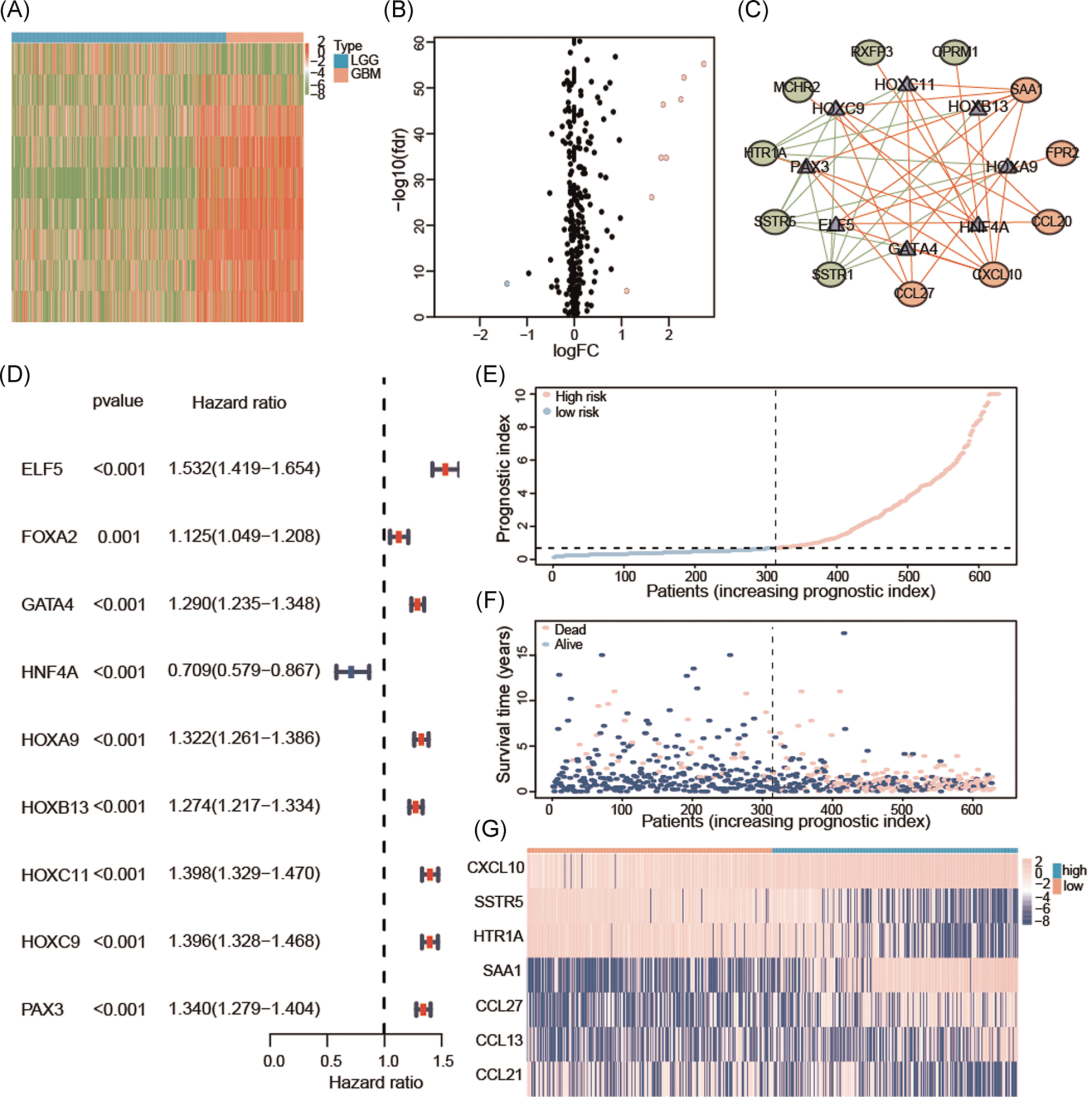
Establishment of TFs‐IRGs regulatory networks and construction of prognostic index based on seven selected IRGs. Differentially expressed TFs were selected using a heatmap (A) and volcano plot (B). The network based on potential regulatory mechanisms between survival‐associated TFs and hub IRGs (C). Forest plot showing the prognostic values of these differentially expressed TFs (D). The distribution of seven IRGs and the prognostic index rank for each patient (E). Survival status of patients in the high‐risk and low‐risk groups (F). Heatmap of the expression levels of seven IRGs (G). IRG, immune‐related genes; TF, transcription factor

To explore the potential molecular mechanism of these hub IRGs in malignancy progress of glioma, we sought to some TFs which could regulate our hub IRGs expression. Out of 318 TFs, only 9 TFs were found differentially expressed in gliomas (Figure 3A,B). All of these TFs were correlated with clinical prognosis, and a forest plot based on TCGA dataset revealed that eight TFs (ELF5, FOXA2, GATA4, HOXA9, HOXB13, HOXC11, HOXC9, and PAX3) were upregulated and 1TFs (HNF4A) was downregulated (Figure 3D). Then we calculated correlation score between survival‐associated TFs and hub IRGs, a correlation score more than 0.4 and *p* < .001 were set as the cut‐off values. Finally, we selected 8 TFs and 11 hub IRGs (5 high risk IRGs and 6 low risk IRGs) to construct the TFs‐IRGs network (Figure 3C).

### 3.4 The prognostic index has good prognostic performance in gliomas and shows significant association with clinicopathological features of gliomas

**Figure 4:**
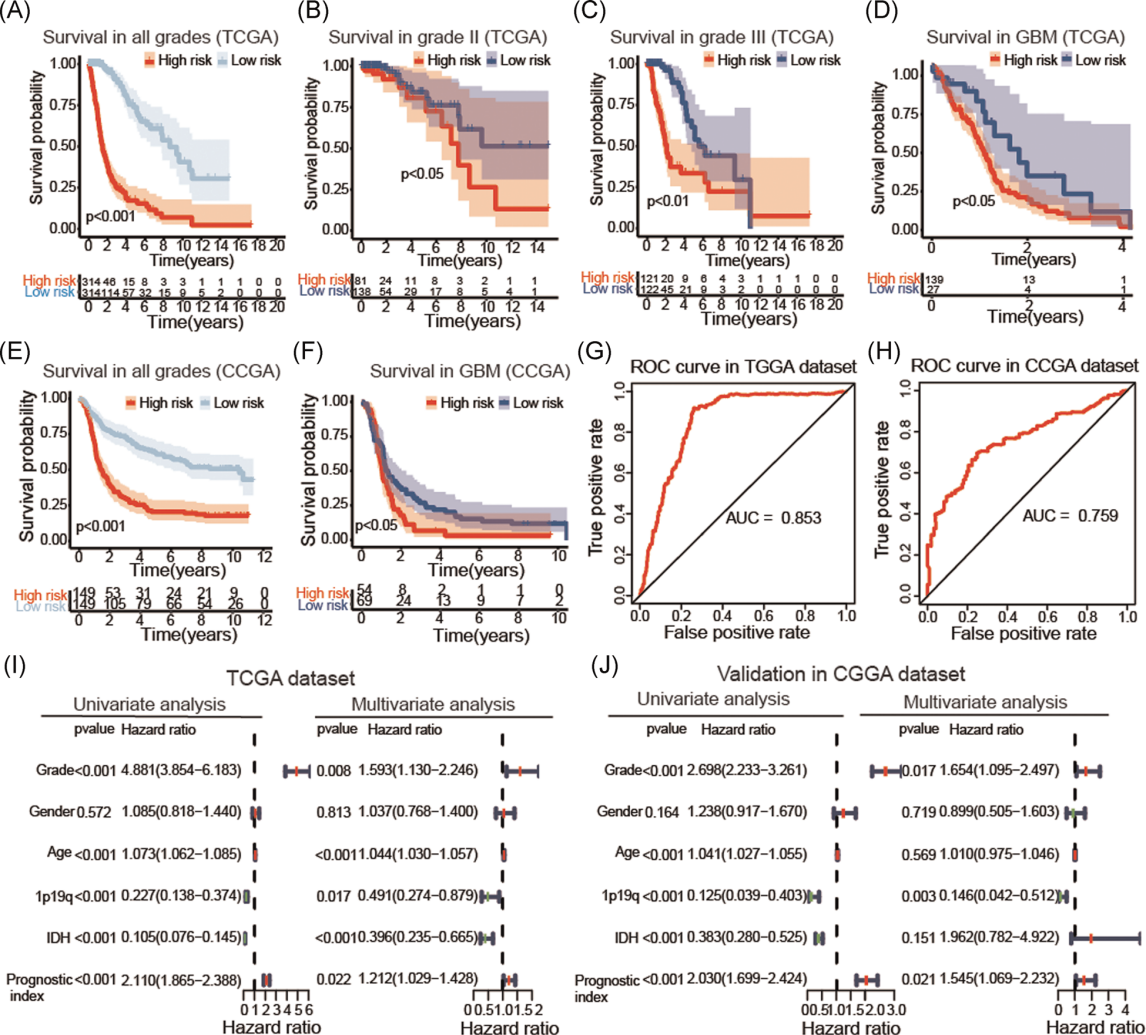
The prognostic value of the prognostic index. Kaplan–Meier survival curves for prognosis prediction using the TCGA datasets (A). Subgroup analysis using Grade II glioma (B), Grade III glioma (C), and GBM (D). Kaplan–Meier survival curves for predicting the clinical prognosis (E) and performed in GBM (F) in the CGGA datasets. ROC curve analysis of the predictive efficiency of our prognostic model in the TCGA (G) and CGGA (H) datasets. Univariate and multiple Cox regression analysis of the association between clinicopathological factors and OS using the TCGA (I) and CGGA (J) datasets. CGGA, Chinese Glioma Genome Atlas; GBM, glioblastoma; OS, overall survival, TCGA, The Cancer Genome Atlas

Seven hub IRGs were selected to construct the prognostic index, and the coefficients were obtained from the LASSO Cox regression algorithm based on the TCGA dataset. Glioma samples were categorized into low‐risk and high‐risk subgroups according to the median prognostic index (Figure 3E–G). There was a clear difference in patient OS between the low‐risk and high‐risk groups across all grade samples (*p* < .001; Figure 4A). The prognostic index also had clinical prognostic value across different grades of gliomas (*p* < .05; WHO Grade II; WHO Grade III; GBM; Figure 4B–D). These conclusions were also validated in the CGGA and GSE16011 dataset (*p* < .05, Figure 4E,F and Figure S1A,B). The area under the ROC curve for clinical outcomes of glioma samples was 0.853 in the TCGA dataset, 0.759 in the CGGA dataset and 0.78 in the GSE16011 dataset, indicating that our prognostic model could accurately predict OS of patients with glioma (Figure 4G,H and Figure S1C).

Univariate Cox regression analysis showed that WHO grade, age, IDH status, 1p/19q status, and prognostic index were strongly associated with the OS of patients based on the TCGA datasets. Multivariate Cox regression analysis revealed that WHO grade (*p* < .01), age (*p* < .01), 1p/19q status (*p* < .05), and prognostic index (*p* < .05) remained significantly correlated with OS (Figure 4I). Similar conclusions were reached using the CGGA and GSE16011 datasets (Figure 4J and Figure S1D). Furthermore, we explored the correlations between the prognostic index and clinicopathological features. The prognostic index was higher in older, 1p/19q non‐codel, IDH‐wildtype, and patients with high‐grade glioma in the TCGA (Figure 6A–E), CGGA (Figure S2) and GSE16011 (Figure S1E–I) datasets, indicating that the prognostic index was related to malignant clinicopathological features in gliomas. Based on the collective findings, our results indicate the prognostic index derived from seven selected IRGs has strong prognostic value in gliomas.

### 3.5 Establishment of a nomogram for predicting clinical prognosis

**Figure 5:**
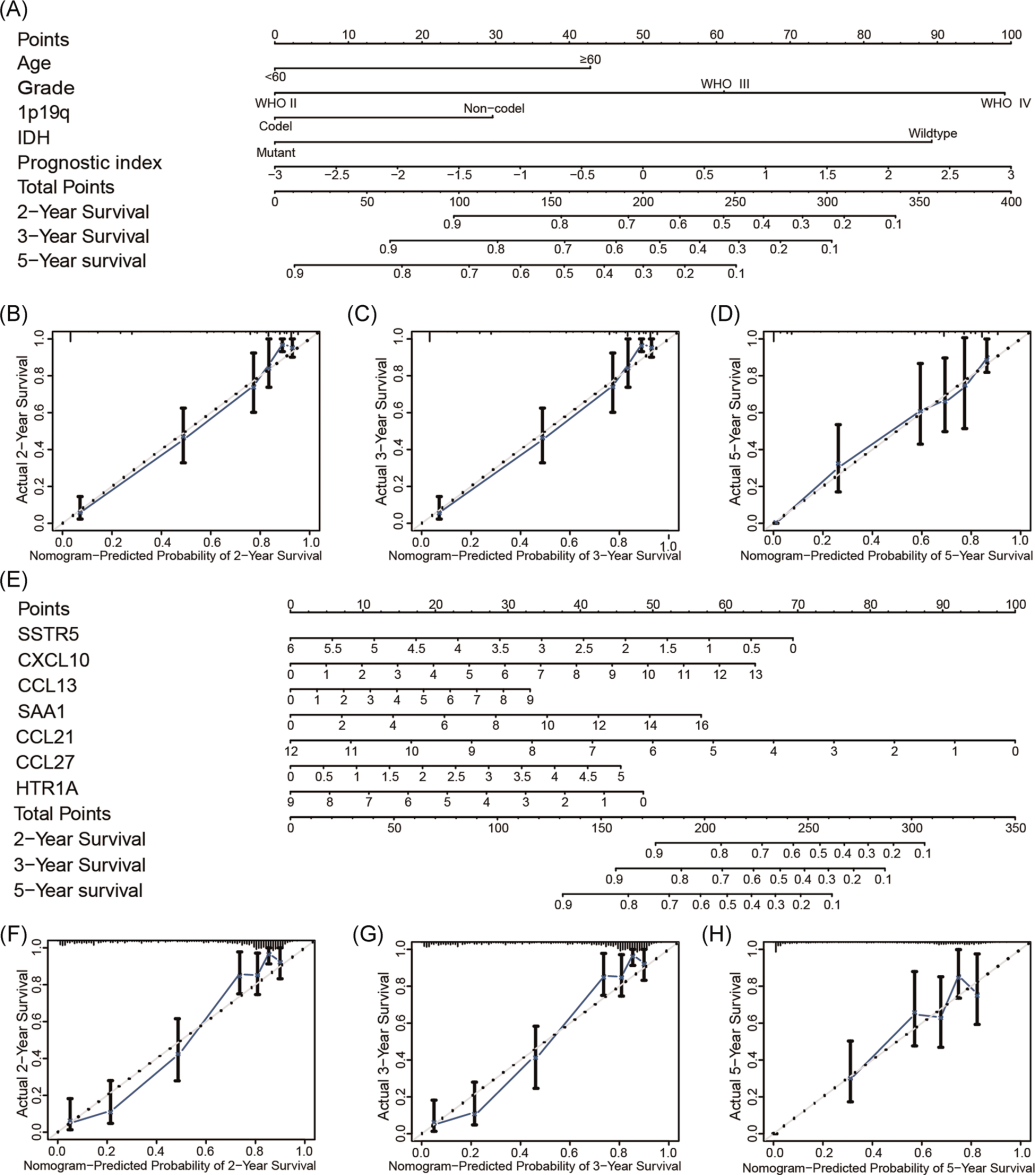
Construction and assessment of nomogram for predicting patients' overall survival. Nomogram based on the clinical characteristics and prognostic index for predicting clinical outcome (A). The calibration curve for predicting clinical outcome at 2 (B), 3 (C), and 5 years (D). Nomogram based on the seven selected IRGs for predicting clinical outcome (E). The calibration curve for predicting clinical outcome at 2 (F), 3 (G), and 5 years (H)

The nomogram integrated age, grade, 1p19q status, IDH status, and prognostic index for gliomas in the TCGA datasets (Figure 5A), and the C‐index was 0.843. The calibration curve for survival probability at 2, 3, and 5 years revealed an optimal agreement between the nomogram prediction and the actual observed outcomes (Figure 5B–D). Also, we integrated the seven IRGs signature to establish a nomogram (Figure 5E), and the C‐index was 0.827. The calibration curve for survival probability at 2, 3, and 5 years showed an optimal agreement between the nomogram prediction and the actual observed outcomes (Figure 5F–H).

### 3.6 The relationships between the prognostic index and infiltration level of six types of immune cells

**Figure 6:**
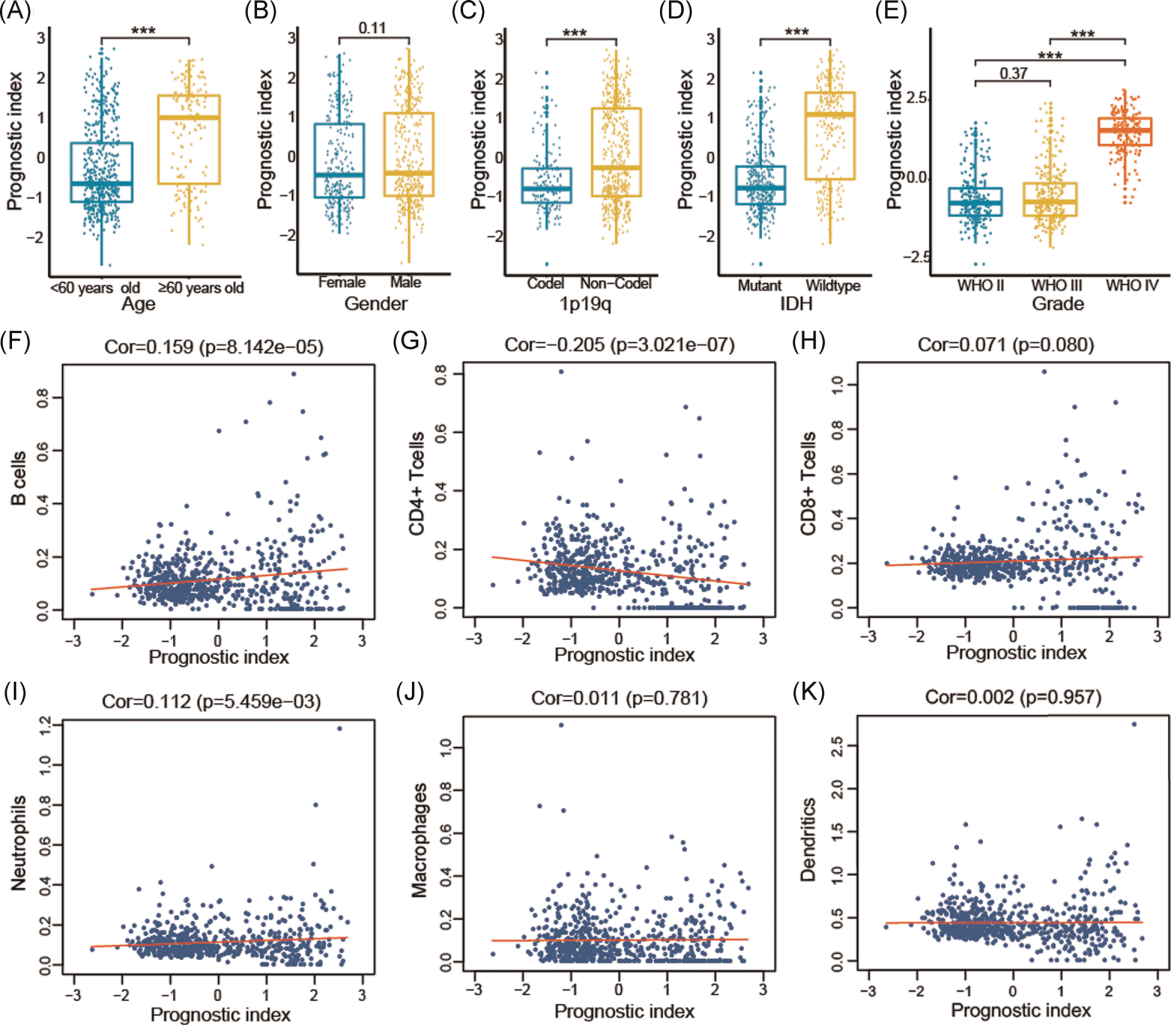
Correlation between prognostic index, clinical characteristics and immune cell infiltration level in the TCGA datasets. Correlation between prognostic index and age (A); gender (B); 1p/19q status (C); IDH status (D); and grade (E). Correlation between the prognostic index and infiltration levels of the six types of immune cells: B cells (F); CD+ 4 T cells (G); CD8+ T cells (H); neutrophils (I); macrophages (J); and dendritic cells (K). *p < .05, **p < .01, ***p < .001. IDH, isocitrate dehydrogenase

To explore whether the prognostic index could reflect the state of the glioma immune microenvironment, the relationships between the prognostic index and infiltration level of six types of immune cells were assessed. The results revealed that prognostic index had positive relationships with B cells (*p* < .001) and neutrophils (*p* < .001), but a negative relationship with CD4+ T cells (*p* < .001), while no significant relationship with CD8+ T cells, macrophages and dendritic cells were observed (Figure 6F–K).

### 3.7 Exploration the role of seven selected IRGs in malignance of glioma

We further explored the correlation between the expression of the seven IRGs and the clinicopathological features of gliomas using the TCGA (Table 1) and CGGA datasets (Table S5). CXCL10, CCL13, SAA1, and CCL27 were more highly expressed in elderly, high‐grade, 1p19q non‐codel, IDH‐wildtype glioma patients, while SSTR5, CCL21, and HTR1A expressions were low in these malignant clinicopathological features of gliomas. The patients with high expression levels of CXCL10, CCL13, SAA1, and CCL27 had a worse OS, while low expression levels of SSTR5, CCL21, HTR1A had a poor prognosis in the TCGA datasets (Figure S3). Gene set enrichment analysis revealed that the JAK/STAT signaling pathway, p53 signaling pathway, and cytokine‐cytokine receptor interaction were most commonly enriched in the CXCL10, CCL13, SAA1, and CCL27 high expression phenotype (Figure S4).

**Table 1:** Relationships between the expression of seven IRGs and the clinicopathological factors of glioma in the TCGA dataset

Genes	Grade (IV/II‐III)		Gender (male/female)		Age (years old) (≥60/<60)		1p19q (noncodel/codel)		IDH (wildtype/mutant)	
	*t*	*p*	*t*	*p*	*t*	*p*	*t*	*p*	*t*	*p*
SSTR5	16.801	<.001	0.374	.708	5.264	<.001	3.6	<.001	8.937	<.001
CXCL10	−21.867	<.001	−1.168	.243	−7.15	<.001	−6.213	<.001	−11.806	<.001
CCL13	−6.43	<.001	−1.366	.172	−2.85	<.001	−2.873	<.001	−5.311	<.001
SAA1	−19.463	<.001	−1.714	.087	−7.822	<.001	−8.771	<.001	−13.266	<.001
CCL21	6.234	<.001	0.982	.327	1.252	.212	1.7385	.084	3.026	.003
CCL27	−9.846	<.001	−2.319	.021	−3.91	<.001	−5.389	<.001	−8.141	<.001
HTR1A	19.106	<.001	0.82	.412	7.262	<.001	4.268	<.001	9.806	<.001

Abbreviations: IRG, immune‐related genes; *p*, *p* value of the Student *t* test; *t*, *t* value of the student test; TCGA, The Cancer Genome Atlas.

### 3.8 The messenger RNA expression of seven selected IRGs in gliomas

**Figure 7:**
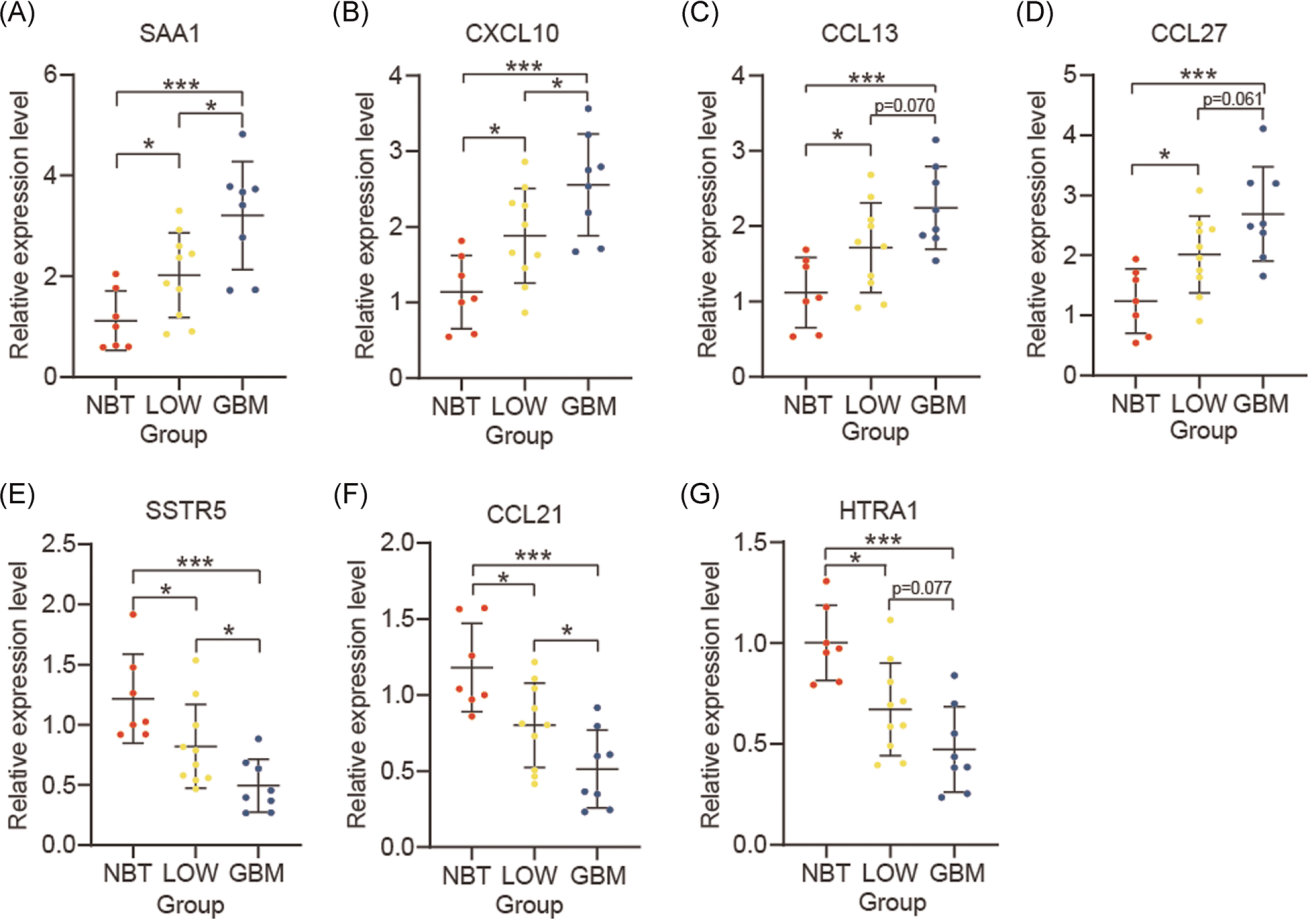
Validation of seven selected hub IRGs by RT‐qPCR. Comparative SAA1 (A), CXCL10 (B), CCL13 (C), CCL27 (D), SSTR5 (E), CCL21 (F) and HTR1A (G) mRNA expression levels in NBT, LGG and GBM. *p < .05, **p < .01, and ***p < .001. GBM, glioblastoma; IRG, immune‐related genes; LGG, low grade glioma; mRNA, messenger RNA; RT‐qPCR, real‐time quantitative polymerase chain reaction

The RT‐qPCR assay indicated that seven IRGs (SAA1, CXCL10, CCL13, CCL27, SSTR5, CCL21, and HTR1A) were differentially expressed between NBT, LGG, and GBM, which were in the main consistent with the results of the bioinformatics analysis (Figure 7A–G).

## 4 DISCUSSION

Despite the significant role of IRGs in tumorigenesis and tumor progression, a systematic, genome‐wide examination of the correlations between molecular mechanisms of IRGs and progression of malignance in gliomas have not been established.^24^, ^25^, ^26^, ^27^, ^28^ In this study, we systematically analyzed IRGs involved in glioma and explored their prognostic value. These results revealed the potential molecular mechanisms of IRGs and the underlying application of IRGs in immunotherapy for glioma treatment. We used the transcriptome profiling data and corresponding clinical information of glioma samples based on the TCGA, CGGA, and GSE16011 datasets to identify differentially expressed and survival‐associated IRGs. We then defined hub IRGs and established a TFs‐IRGs network. We constructed a prognostic model derived from seven selected hub IRGs, which had good prognostic performance and showed significant association with clinicopathological features of gliomas and immune cell infiltration levels. We also constructed a prognostic nomogram for predicting patients' OS.

Chemokines play a crucial role in the innate immune system. The main molecular functions of chemokines are to control the localization and migration of inflammatory cells and to initiate the adaptive immune response, thereby promoting tumor malignance and metastasis.^29^, ^30^ The chemokine system can promote malignance in glioma, which is related to chemotaxis, inflammatory infiltration, metastasis, and proliferation.^31^, ^32^ Our functional enrichment analysis indicated that enrichment of differentially expressed IRGs was involved in cytokine‐cytokine receptor interaction and chemokine signaling pathways. Together, these results indicated that IRGs could play a significant role in the malignant progression of gliomas by influencing the chemokine system and some immune‐related pathways.

To comprehensively analyze the underlying biological functions of IRGs in malignance progression glioma, we explored the mechanisms by which crucial TFs regulated hub IRGs through constructing a TFs‐IRGs regulatory network. Eight survival‐associate TFs occupied significant positions in our network. Our TFs‐mediated networks are expected to guide future analyses of molecular mechanisms of malignancy in gliomas. PAX3 was upregulated in glioma cells, and was associated with a poorer prognosis for patients. Moreover, PAX3 could facilitate cell proliferation and invasion, inhibit apoptosis, and played an oncogenic role in gliomas through promoting proliferation.^33^ Some studies have reported that suppressed GATA4 and HOXA9 expression can inhibit malignancy and metastasis in glioma. However, the molecular mechanisms of HOXC11, HOXC9, ELF5, and HNF4A function in the development and progression of gliomas remain unclear and require further exploration in the future.^34^, ^35^, ^36^

We used a prognostic model derived from seven hub IRGs to ascertain whether IRGs expression was monitoring the immune microenvironment and an independent prognostic index for diagnosis of gliomas. Our results showed that poor OS was strongly associated with the high‐risk subgroup in the TCGA, CGGA, and GSE16011 datasets. We additionally observed a significant correlation between the prognostic index and malignant clinical characteristics of gliomas. Furthermore, our prognostic index could serve as an independent prognostic index for diagnosis of glioma, as determined by univariate and multivariate Cox regression analyses using the TCGA, CGGA, and GSE16011 datasets. Subsequently, we constructed a nomogram for predicting patients' OS. The C‐index and calibration curve for survival prediction showed that our nomogram was reliable. The results further indicated that the prognostic index and the seven selected IRGs had clinical prognostic value in gliomas.

We explored the role that seven selected IRGs played in malignance in glioma, and found that IRGs expression levels were significantly correlated with malignant clinicopathological features of gliomas, immune‐related biological processes, and cancer‐related signaling pathways. In previous studies, the molecular mechanisms of HTR1A, CCL13, CCL21, and CCL27 in gliomas remained unclear. Barbieri et al. indicated that high SSTR5 expression levels could inhibit growth in gliomas,^37^ and Maru et al.^38^ showed high CXCL10 expression could promote malignancy in glioma. Knebel et al.^39^ demonstrated that SAA1 had dual effects on glioma migration and invasiveness in different human glioma cell lines. There is little information on the molecular mechanisms of the seven selected IRGs in malignancy in glioma to date.

We also characterized the immune infiltration landscape to analyze tumor‐immune interactions. The correlations between the prognostic index and infiltration levels of six immune cell types were determined to explore the regulatory mechanisms of the immune microenvironment in gliomas. Our results showed that the prognostic index was positively correlated with B cells and neutrophils, and negatively correlated with CD4+ T cells. The immune cells actively participate in the progression of malignance in glioma, and our results indicate that the prognostic index can serve as a tool for monitoring the level of immune cell infiltration. The molecular mechanism of immune cells in the progression of malignancy in gliomas has not yet been systematically analyzed. Ge et al.^40^ indicated that patients with gliomas had higher CD4+ cells infiltration levels than healthy patients. Moreover, Sokratous et al.^41^ indicated that patients with gliomas and high cytotoxic T cell infiltration levels had poor OS.

Nonetheless, there are some limitations in this study. First, the samples did not contain information about extent of tumor resection which connected to prognosis in patients with glioma, a collection of more detailed clinical information was needed to be carried in the future. Second, the number of local tissues used in RT‐qPCR assay are little, we need perform more in vitro or in vivo experiments to validate our bioinformatics results.

## 5 CONCLUSION

In conclusion, we have comprehensively investigated the expression patterns, prognostic value, and potential functions of IRGs in gliomas. IRGs expression levels correlated with malignant clinical characteristics, cancer‐related biological processes, and signaling pathways associated with malignancy progression. Our results also demonstrate that the prognostic index plays a crucial role in the malignancy in glioma, and will enable the design of immune checkpoint inhibitors for the successful implementation of immunotherapy in gliomas.

## CONFLICT OF INTERESTS

The authors declare that there are no conflict of interests.

## AUTHOR CONTRIBUTIONS

Project design and data acquisition: Haitao Luo Chuming Tao. Writing the manuscript: Peng Wang. Statistical comparison: Jingying Li. Drafting the manuscript and contributing to revision: Kai Huang and Xingen Zhu. All authors edited and approved the final version of manuscript.

## Supporting information

Supporting information.

Supporting information.

Supporting information.

Supporting information.

Supporting information.

Supporting information.

Supporting information.

Supporting information.

Supporting information.

## Data Availability

We obtained glioma clinicopathological data from the TCGA, CGGA, and GSE16011 datasets. The IRGs data were obtained from the ImmPort dataset. We obtained copy number alteration data from cBioPortal. A list of 318 TFs was obtained from the Cistrome Cancer dataset. We obtained immune cells infiltration level data from the TIMER dataset.
