# Supplementary figures and images for: Development of a prognostic index based on immunogenomic landscape analysis in glioma
Source: Immun Inflamm Dis. 2021 Jan 27;9(2):467–79. doi: 10.1002/iid3.407 (PMC8127549; doi:10.1002/iid3.407)

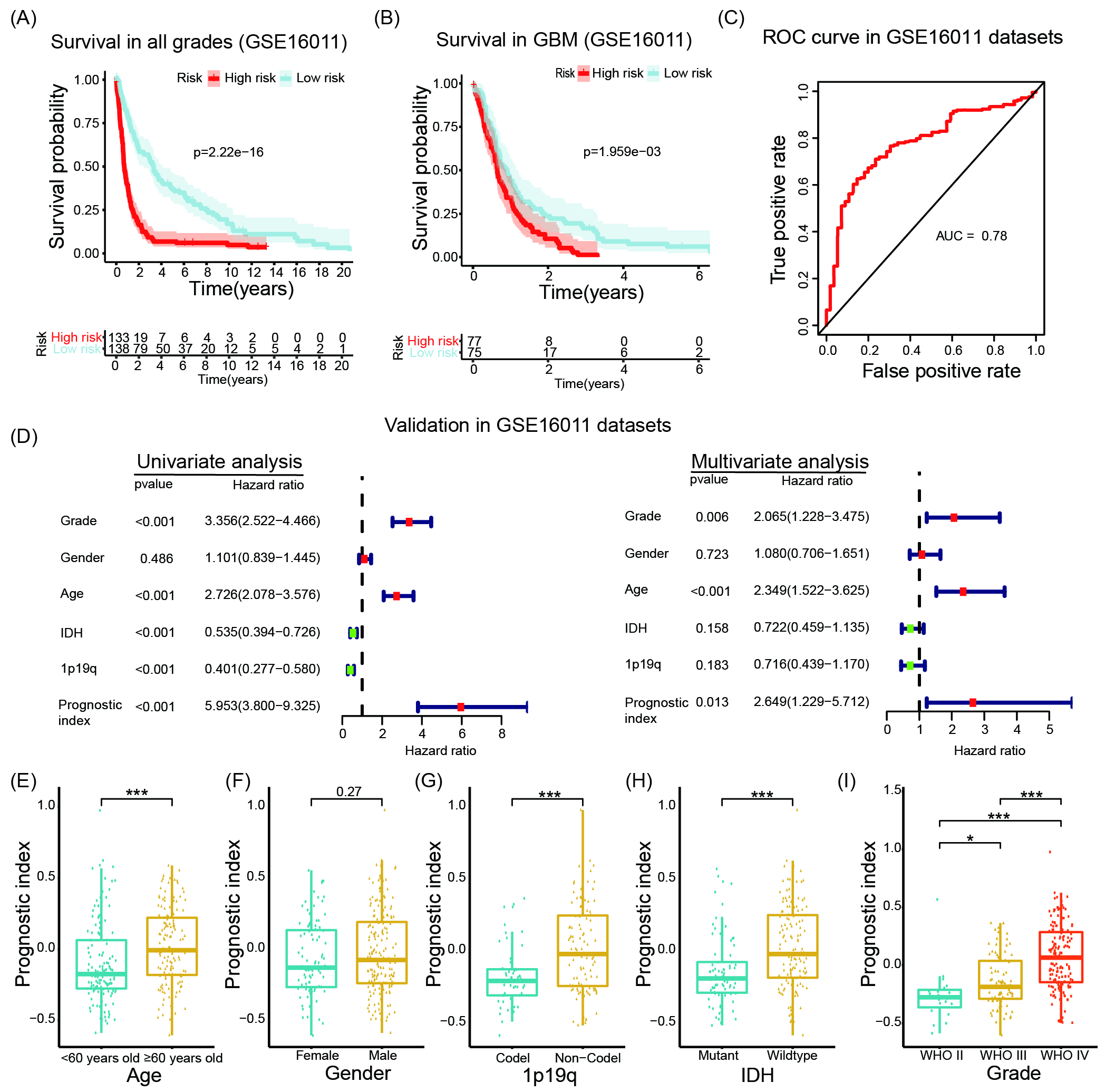

Supplement: Supplementary file 1 — Supporting information. [file IID3-9-467-s002.tif]

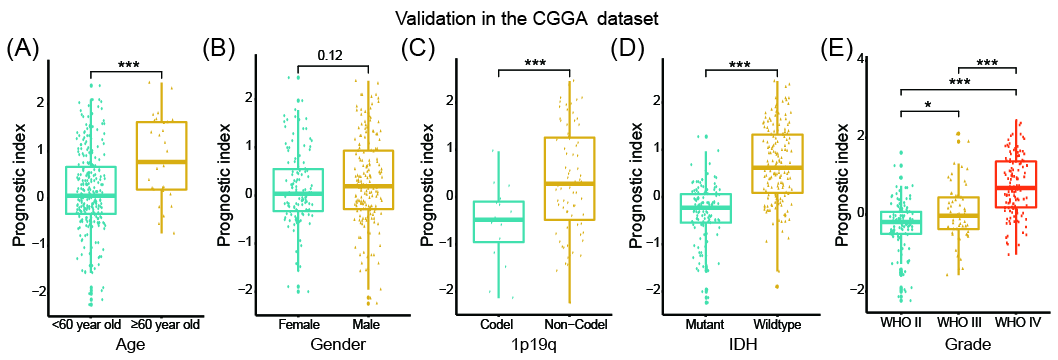

Supplement: Supplementary file 2 — Supporting information. [file IID3-9-467-s004.tif]

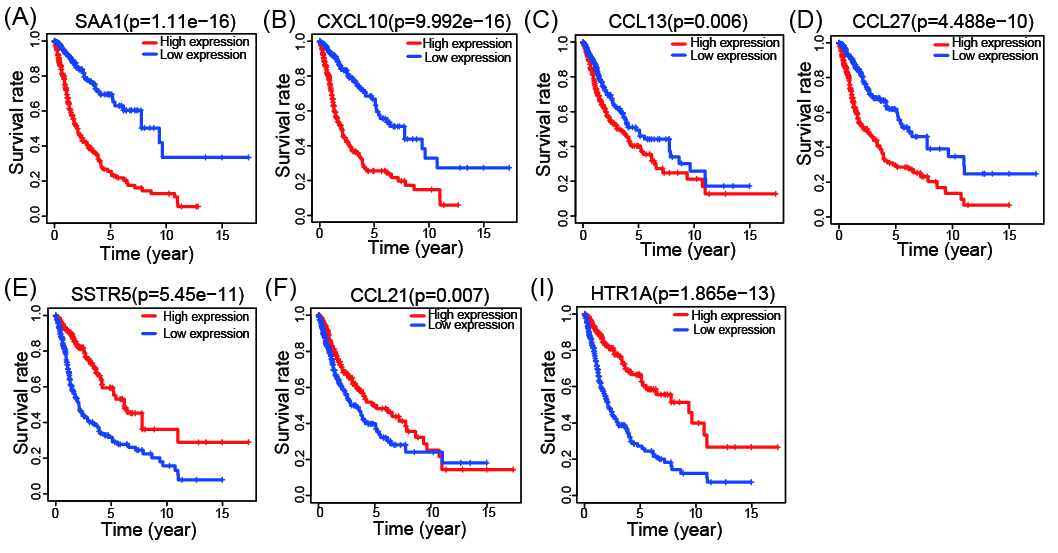

Supplement: Supplementary file 3 — Supporting information. [file IID3-9-467-s003.tif]

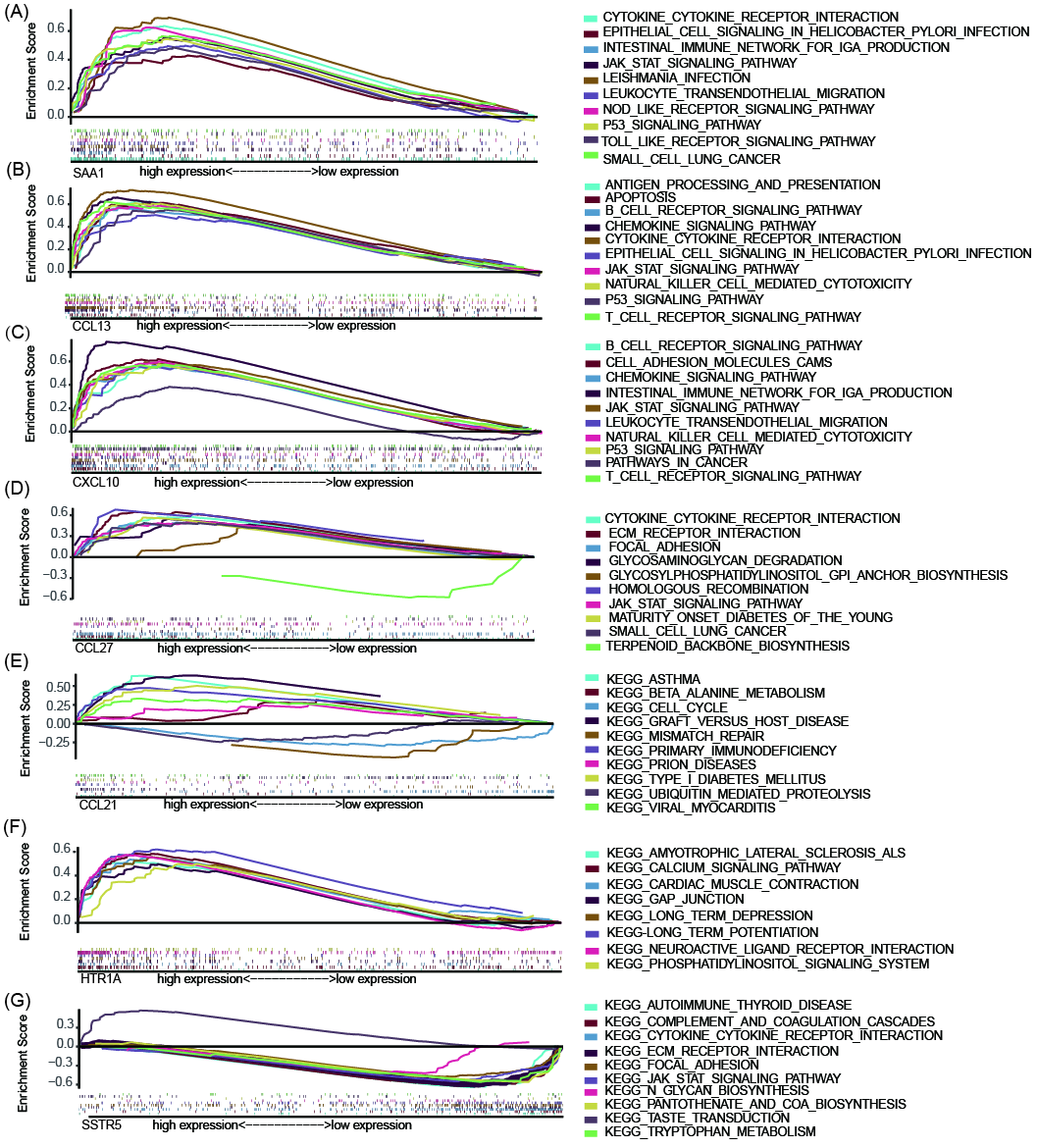

Supplement: Supplementary file 4 — Supporting information. [file IID3-9-467-s009.tif]
