# Supplementary material for: Development of a prognostic index based on immunogenomic landscape analysis in glioma
Source: Immun Inflamm Dis. 2021 Jan 27;9(2):467–79. doi: 10.1002/iid3.407 (PMC8127549; doi:10.1002/iid3.407)
Supplement: Supplementary file 5 — Supporting information. [file IID3-9-467-s001.docx]

**TABLE S1** Clinicopathological features of patients included in this study

|  |  | **TCGA dataset** | | **CGGA dataset** | | **GSE16011 dataset** | |
| --- | --- | --- | --- | --- | --- | --- | --- |
|  |  | **Number** | **Percentage** | **Number** | **Percentage** | **Number** | **Percentage** |
| **Total** |  | 628 | 100% | 298 | 100% | 263 | 100% |
| **Age** |  | 14-89 (47) |  | 12-70 (42) |  | 14-81  (51) |  |
|  | ＜median | 308 | 49.04% | 145 | 48.66% | 133 | 50.57% |
|  | ≥median | 320 | 50.96% | 151 | 50.67% | 129 | 49.05% |
|  | NA | 0 | 0% | 2 | 0.67% | 1 | 0.38% |
| **Gender** | Female | 266 | 42.36% | 121 | 40.60% | 87 | 33.08% |
|  | Male | 362 | 51.91% | 177 | 59.40% | 176 | 66.92% |
|  | NA | 0 | 0% | 0 | 0% | 0 | 0% |
| **Grade** |  |  |  |  |  |  |  |
|  | WHO II | 219 | 34.87% | 115 | 38.59% | 23 | 8.74% |
|  | WHO III | 243 | 38.69% | 57 | 19.13% | 85 | 32.32% |
|  | WHO IV | 156 | 24.84% | 123 | 41.28% | 155 | 58.94% |
|  | NA | 0 | 0% | 3 | 1.01% | 0 | 0% |
| **IDH** |  |  |  |  |  |  |  |
|  | Wildtype | 235 | 37.42% | 164 | 55.03% | 131 | 49.81% |
|  | Mutation | 384 | 61.15% | 132 | 44.30% | 78 | 29.66% |
|  | NA | 9 | 1.43% | 2 | 0.67% | 54 | 20.53% |
| **1p/19q** |  |  |  |  |  |  |  |
|  | Non-codel | 470 | 74.84% | 75 | 25.17% | 103 | 39.16% |
|  | Codel | 152 | 24.20% | 16 | 5.37% | 53 | 20.15% |
|  | NA | 6 | 0.96% | 207 | 69.46% | 107 | 40.69% |
