# Supplementary material for: Development of a prognostic index based on immunogenomic landscape analysis in glioma
Source: Immun Inflamm Dis. 2021 Jan 27;9(2):467–79. doi: 10.1002/iid3.407 (PMC8127549; doi:10.1002/iid3.407)
Supplement: Supplementary file 6 — Supporting information. [file IID3-9-467-s006.docx]

**TABLE S2** Clinical data of the patients that used in the RT-qPCR

| **patient number** | **Age**  **(years old)** | **Gender** | **paired/**  **unpaired** | **Grade of**  **glioma** | **Hospitalization date** |
| --- | --- | --- | --- | --- | --- |
| 1074273 | 47 | Male | unpaired | NBT | 2019.05.25 |
| 909765 | 72 | Female | unpaired | NBT | 2018.09.12 |
| 853216 | 67 | Male | unpaired | NBT | 2018.03.12 |
| 813723 | 38 | Male | unpaired | NBT | 2018.02.19 |
| 742367 | 53 | Female | unpaired | NBT | 2017.11.02 |
| 651479 | 62 | Female | unpaired | NBT | 2017.07.08 |
| 623468 | 71 | Male | unpaired | NBT | 2017.04.29 |
| 1134567 | 65 | Female | unpaired | LGG | 2019.09.17 |
| 1093421 | 54 | Male | unpaired | LGG | 2019.07.12 |
| 989535 | 35 | Female | unpaired | LGG | 2019.01.27 |
| 919432 | 71 | Female | unpaired | LGG | 2018.10.14 |
| 853419 | 43 | Male | unpaired | LGG | 2018.03.28 |
| 806710 | 28 | Male | unpaired | LGG | 2018.02.02 |
| 801328 | 58 | Male | unpaired | LGG | 2018.01.23 |
| 768913 | 46 | Female | unpaired | LGG | 2017.11.29 |
| 701387 | 61 | Female | unpaired | LGG | 2017.09.27 |
| 639871 | 56 | Male | unpaired | LGG | 2017.05.18 |
| 1223459 | 39 | Male | unpaired | GBM | 2019.12.21 |
| 1034975 | 47 | Female | unpaired | GBM | 2019.02.17 |
| 985412 | 57 | Male | unpaired | GBM | 2019.01.07 |
| 921748 | 55 | Female | unpaired | GBM | 2018.11.28 |
| 864190 | 68 | Male | unpaired | GBM | 2018.04.19 |
| 713891 | 49 | Female | unpaired | GBM | 2017.10.14 |
| 663917 | 71 | Male | unpaired | GBM | 2017.07.27 |
| 610438 | 64 | Male | unpaired | GBM | 2017.02.13 |
