# Supplementary material for: Development of a prognostic index based on immunogenomic landscape analysis in glioma
Source: Immun Inflamm Dis. 2021 Jan 27;9(2):467–79. doi: 10.1002/iid3.407 (PMC8127549; doi:10.1002/iid3.407)
Supplement: Supplementary file 7 — Supporting information. [file IID3-9-467-s007.docx]

**TABLE S3** Primer sequences used for Realtime PCR analysis

| **Gene symbol** | **Gen Bank Accession no.** | **Primer set sequence (5’->3’)** | **Amplicon size (bp)** |
| --- | --- | --- | --- |
| SAA1 | NM_00331.6 | Forward:  TCAGGTGAGGAGCACACCAA  Reverse:  CATGTCCCGAGCCCCATCAA | 260 |
| CXCL10 | NM_001565.4 | Forward:  CCACGTGTTGAGATCATTGCT  Reverse:  TGCATCGATTTTGCTCCCCT | 152 |
| CCL13 | NM_005408.3 | Forward:  TGCAGAGGCTGAAGAGCTATG  Reverse:  CCCAGTTTGGTTCTGAAGATGA | 76 |
| CCL27 | NM_006664.4 | Forward:  AGGAAGAGTCTAGGCTGAGCA  Reverse:  GGCAGTAGGAATGCTGCTGTA | 106 |
| SSTR5 | NM_001172560.1 | Forward:  GGCTCCCCCAGCCTGA  Reverse:  CACGTAGATGACCAGCGTGT | 228 |
| CCL21 | NM_002989.4 | Forward:  GCTCTGGCCTCTTACTCACC  Reverse:  CTCCATCACTGCCTTGGGTC | 112 |
| HTRA1 | NM_002775.5 | Forward:  ACCGACAGGCCAAAGGAAAA  Reverse:  GCTCCTGAGATCACGTCTGG | 130 |
