# Supplementary material for: Development of a prognostic index based on immunogenomic landscape analysis in glioma
Source: Immun Inflamm Dis. 2021 Jan 27;9(2):467–79. doi: 10.1002/iid3.407 (PMC8127549; doi:10.1002/iid3.407)
Supplement: Supplementary file 8 — Supporting information. [file IID3-9-467-s005.docx]

**TABLE S4** General characteristics of 91 survival-associated IRGs

| **Genes** | **P-value** | **HR** | **HR.95L** | **HR.95H** |
| --- | --- | --- | --- | --- |
| GDF15 | 6.98E-44 | 1.513544 | 1.427586 | 1.604677 |
| SAA1 | 1.44E-43 | 1.265704 | 1.224169 | 1.308648 |
| SAA2 | 1.41E-40 | 1.293398 | 1.245411 | 1.343235 |
| PLA2G2A | 2.24E-37 | 1.224147 | 1.186749 | 1.262724 |
| SSTR1 | 1.17E-34 | 0.719623 | 0.682805 | 0.758427 |
| TNFSF14 | 2.64E-34 | 1.63927 | 1.514273 | 1.774585 |
| MMP9 | 3.04E-34 | 1.293991 | 1.241515 | 1.348685 |
| ULBP3 | 3.40E-33 | 1.685618 | 1.547868 | 1.835627 |
| DES | 4.06E-33 | 1.31902 | 1.260645 | 1.380099 |
| FCGR2B | 1.45E-32 | 1.418464 | 1.338993 | 1.502653 |
| FAM19A3 | 2.96E-32 | 1.501629 | 1.403763 | 1.606319 |
| CXCL10 | 4.03E-32 | 1.367989 | 1.298596 | 1.441089 |
| LTF | 1.25E-31 | 1.199051 | 1.163141 | 1.236069 |
| STC1 | 5.00E-31 | 1.414695 | 1.334044 | 1.500221 |
| KLRC2 | 6.98E-31 | 0.796465 | 0.766306 | 0.827811 |
| PRLHR | 1.19E-30 | 0.729805 | 0.691688 | 0.770021 |
| ESM1 | 2.85E-28 | 1.331408 | 1.265362 | 1.400902 |
| PI3 | 3.86E-28 | 1.245778 | 1.197936 | 1.295531 |
| CCL20 | 4.38E-28 | 1.395548 | 1.314999 | 1.481031 |
| IL2RA | 5.68E-27 | 1.328156 | 1.261206 | 1.398659 |
| IL13RA2 | 8.86E-27 | 1.304406 | 1.242504 | 1.369393 |
| SSTR5 | 1.93E-26 | 0.493865 | 0.433682 | 0.5624 |
| CCL7 | 2.69E-26 | 1.474768 | 1.372634 | 1.584503 |
| IL17B | 2.82E-26 | 1.605637 | 1.471098 | 1.75248 |
| CD70 | 2.34E-25 | 1.317869 | 1.251104 | 1.388198 |
| KLRC4 | 2.86E-25 | 0.712869 | 0.668762 | 0.759884 |
| RETN | 3.84E-25 | 1.617564 | 1.476865 | 1.771667 |
| IDO1 | 1.23E-23 | 1.3144 | 1.24597 | 1.386589 |
| UCN2 | 1.69E-23 | 1.51212 | 1.394288 | 1.639909 |
| IL1R2 | 6.97E-23 | 1.382679 | 1.29633 | 1.474779 |
| FPR2 | 7.37E-23 | 1.428822 | 1.330815 | 1.534048 |
| CCR4 | 1.14E-22 | 1.621265 | 1.471899 | 1.785789 |
| CD3D | 3.50E-22 | 1.424807 | 1.326294 | 1.530637 |
| ICOS | 8.69E-22 | 1.580142 | 1.439107 | 1.734997 |
| LBP | 2.97E-21 | 1.326603 | 1.251183 | 1.40657 |
| CCL27 | 1.80E-20 | 1.908808 | 1.665038 | 2.188267 |
| EREG | 2.15E-20 | 1.414634 | 1.314436 | 1.522469 |
| IL1F8 | 6.87E-20 | 1.597295 | 1.444516 | 1.766232 |
| AREG | 7.98E-20 | 1.349109 | 1.264965 | 1.43885 |
| CER1 | 9.20E-20 | 1.562209 | 1.419066 | 1.719791 |
| HTR1A | 9.89E-20 | 0.662729 | 0.606476 | 0.7242 |
| GLP1R | 4.57E-18 | 0.694197 | 0.639178 | 0.753953 |
| EDN2 | 9.59E-18 | 1.492618 | 1.362094 | 1.635649 |
| GDNF | 1.29E-17 | 0.599223 | 0.53281 | 0.673913 |
| ELANE | 1.61E-17 | 1.609338 | 1.442457 | 1.795526 |
| IL24 | 2.27E-17 | 1.446915 | 1.32848 | 1.575908 |
| TNFSF11 | 2.86E-17 | 1.837435 | 1.595671 | 2.115829 |
| XCL1 | 1.74E-16 | 1.711456 | 1.506084 | 1.944834 |
| CCL23 | 1.80E-16 | 1.605725 | 1.434556 | 1.797317 |
| XCL2 | 2.21E-16 | 1.69343 | 1.493324 | 1.92035 |
| CXCL6 | 4.64E-16 | 1.28345 | 1.208429 | 1.363128 |
| S100A12 | 5.12E-16 | 1.394421 | 1.286742 | 1.51111 |
| GALR3 | 1.50E-15 | 0.463642 | 0.383848 | 0.560024 |
| FAM3D | 3.79E-15 | 1.629105 | 1.442478 | 1.839876 |
| CCR3 | 6.34E-15 | 1.977612 | 1.666094 | 2.347375 |
| GAL | 7.92E-15 | 1.285048 | 1.206258 | 1.368983 |
| CAMP | 8.36E-15 | 1.297285 | 1.214768 | 1.385408 |
| PROK2 | 1.42E-14 | 1.408352 | 1.290715 | 1.53671 |
| LEP | 1.50E-14 | 1.643839 | 1.448187 | 1.865923 |
| CCL13 | 5.50E-14 | 1.321537 | 1.22891 | 1.421146 |
| CCR8 | 1.56E-13 | 1.912434 | 1.60998 | 2.271706 |
| CCL26 | 2.50E-13 | 1.398491 | 1.27836 | 1.52991 |
| CELA1 | 3.23E-13 | 1.621474 | 1.42374 | 1.84667 |
| NPPB | 2.17E-12 | 1.352368 | 1.243113 | 1.471225 |
| BMP5 | 4.77E-12 | 1.249629 | 1.17311 | 1.331138 |
| PPP3R2 | 5.84E-12 | 0.303382 | 0.216021 | 0.426072 |
| PMCH | 7.63E-11 | 1.398205 | 1.263945 | 1.546727 |
| TNFRSF9 | 6.46E-10 | 1.442208 | 1.284051 | 1.619845 |
| IL31RA | 6.49E-10 | 1.312371 | 1.203935 | 1.430573 |
| SPINK5 | 7.04E-10 | 0.499597 | 0.40069 | 0.62292 |
| CHP2 | 9.77E-10 | 0.619988 | 0.531885 | 0.722685 |
| LPA | 1.07E-09 | 0.513944 | 0.414968 | 0.636528 |
| C20orf186 | 1.55E-09 | 1.33574 | 1.21596 | 1.46732 |
| SCT | 3.63E-09 | 0.621503 | 0.530676 | 0.727875 |
| CCL18 | 4.01E-09 | 1.156619 | 1.101897 | 1.214059 |
| PF4V1 | 8.04E-09 | 1.529789 | 1.324003 | 1.76756 |
| CSF3 | 7.16E-08 | 1.167526 | 1.103552 | 1.235209 |
| GDF7 | 7.54E-08 | 0.642742 | 0.547108 | 0.755092 |
| CD1A | 2.64E-07 | 1.289154 | 1.170316 | 1.420061 |
| GLP2R | 2.74E-07 | 0.748757 | 0.670546 | 0.836091 |
| MMP12 | 3.32E-06 | 1.167679 | 1.093822 | 1.246523 |
| MCHR2 | 3.66E-05 | 0.851045 | 0.7883 | 0.918783 |
| HTR3B | 6.39E-05 | 0.777612 | 0.6874 | 0.879663 |
| RXFP3 | 8.39E-05 | 0.667929 | 0.546244 | 0.816721 |
| IL1F9 | 9.95E-05 | 1.463381 | 1.208047 | 1.772683 |
| IAPP | 0.000143686 | 0.568319 | 0.424691 | 0.76052 |
| IFNA21 | 0.000148293 | 0.516922 | 0.367603 | 0.726896 |
| HNF4A | 0.000804176 | 0.708542 | 0.579236 | 0.866714 |
| CCL21 | 0.000896683 | 0.744275 | 0.625225 | 0.885995 |
| OPRM1 | 0.002009915 | 0.753768 | 0.629999 | 0.901854 |
| LCN15 | 0.007694509 | 0.820461 | 0.709344 | 0.948985 |
