# Supplementary material for: Development of a prognostic index based on immunogenomic landscape analysis in glioma
Source: Immun Inflamm Dis. 2021 Jan 27;9(2):467–79. doi: 10.1002/iid3.407 (PMC8127549; doi:10.1002/iid3.407)
Supplement: Supplementary file 9 — Supporting information. [file IID3-9-467-s008.docx]

**TABLE S5** Relationship between the expression of the seven IRGs and the clinical characteristics of gliomas in the CGGA dataset

| **Genes** | **Grade**  **(IV/ II-III)** | | **Gender**  **(Male/ Female)** | | **Age (years old)**  **(≥60/＜60)** | | **1p/19q**  **(Non-Codel/ Codel)** | | **IDH**  **(Wildtype/ Mutant)** | |
| --- | --- | --- | --- | --- | --- | --- | --- | --- | --- | --- |
|  | **t** | **P** | **t** | **P** | **t** | **P** | **t** | **P** | **t** | **P** |
| SSTR5 | 2.174 | 0.031 | 0.533 | 0.594 | 1.472 | 0.149 | 1.763 | 0.092 | 3.754 | <0.001 |
| CXCL10 | -8.969 | <0.001 | -0.973 | 0.331 | -3.888 | <0.001 | -3.932 | <0.001 | -9.059 | <0.001 |
| CCL13 | -3.707 | <0.001 | -0.496 | 0.620 | -1.144 | 0.262 | -1.714 | 0.096 | -3.292 | <0.001 |
| SAA1 | -10.136 | <0.001 | -1.901 | 0.058 | -2.502 | 0.018 | -4.242 | <0.001 | -9.658 | <0.001 |
| CCL21 | 1.471 | 0.143 | 0.401 | 0.689 | 1.037 | 0.307 | -2.89 | 0.008 | 0.822 | 0.412 |
| CCL27 | -7.341 | <0.001 | -0.676 | 0.500 | -2.712 | 0.011 | -4.199 | <0.001 | -12.21 | <0.001 |
| HTR1A | 3.344 | <0.001 | 0.334 | 0.738 | 3.507 | <0.001 | 0.866 | 0.396 | 3.489 | <0.001 |

t: t value of student’s test; P: *P*-value of student’s t test.
